# Phytoremediation of a Highly Arsenic Polluted Site, Using *Pteris vittata* L. and Arbuscular Mycorrhizal Fungi

**DOI:** 10.3390/plants9091211

**Published:** 2020-09-16

**Authors:** Simone Cantamessa, Nadia Massa, Elisa Gamalero, Graziella Berta

**Affiliations:** Department of Sciences and Technological Innovation, University of Piemonte Orientale, viale T. Michel, 11-15121 Alessandria, Italy; nadia.massa@uniupo.it (N.M.); elisa.gamalero@uniupo.it (E.G.); graziella.berta@uniupo.it (G.B.)

**Keywords:** phytoremediation, arsenic, *Pteris vittata* L., arbuscular mycorrhizal fungi

## Abstract

Phytoremediation is a promising green technique for the restoration of a polluted environment, but there is often a gap between lab and field experiments. The fern, *Pteris vittata* L., can tolerate a high soil arsenic concentration and rapidly accumulate the metalloid in its fronds. Arbuscular mycorrhizal fungi (AMF) are mutualistic fungi that form a symbiosis with most land plants’ roots, improve their growth, and induce stress tolerance. This paper reports the results obtained using *P. vittata* inoculated with AMF, to extract Arsenic (As) from an industrial site highly contaminated also by other pollutants. Two experiments have been performed. In the first one, AMF colonized ferns were grown for two years under controlled conditions in soil coming from the metallurgic site. Positive effects on plant health and As phytoextraction and accumulation were detected. Then, considering these results, we performed a three year in situ experiment in the industrial site, to assess the remediation of As at two different depths. Our results show that the colonization of *P. vittata* with AMF improved the remediation process of As with a significant impact on the depth 0–0.2 m.

## 1. Introduction

Arsenic (As) is a metalloid included in the V group of the periodic table. It is often considered as heavy metal and in the environment is probably more metallic than non-metallic [1]. It is remarked as one of the top health hazards and linked to bladder, lung, skin, and prostate cancers [2]. Anthropogenic activities such as the disposal of industrial chemical waste, mining [3], the residue of As deriving from the metallurgic processes melting copper, gold, lead and zinc, the combustion of fossil fuels, the production of glass handicrafts [4], the use of compost and organic feed, the electronics industry, pharmaceutical preparations [5], and agriculture [6] have contributed to an exponential increase in the amount of As in the environment. It has been shown that As can enter the food chain: rice, which is the staple food for around 50% of the world’s population, can accumulate As more efficiently than other cereals [7] and the accumulation capability is related to cultivar [8,9]. Meharg and co-authors [10] reported that one in 10,000 Italian population will suffer cancer due to exposure of As from rice consumption.

Remediation of As contaminated soils could be carried out with chemical and physical methods. However, these treatments are expensive and can modify the soil properties, reducing biodiversity, and, in many cases, not allowing plant growth. Green technologies, such as phytoextraction or phytoimmobilization, are cheaper techniques that do not affect soil fertility and biodiversity [11,12]. Several studies highlight *Pteris vittata* L. as one of the best candidates for As phytoremediation [13,14,15,16,17]. This fern can tolerate up to 1500 ppm As in soil and shows a fast accumulation of this metalloid in its fronds. Moreover, it is characterized by a vigorous growth with large biomass, an extensive root system development, and a high growth rate. These qualities make *P. vittata* an ideal plant for As remediation purposes. However, as shown by proteomic analyses, its tolerance to As is not complete. In fact, in the fronds, As induces the down-regulation of enzymes involved in sugar metabolism, bioenergetics, photosynthesis, and carbon fixation [18]. In addition, As polluted soils generally contain many other contaminants, among which heavy metals (HM), that can affect plant growth [19].

Arbuscular mycorrhizal fungi (AMF) are symbiotic microscopic fungi belonging to the Glomeromycotina, subphylum of phylum Mucoromycota [20], able to positively influence plant performance in many ways, including by enhancement of heavy metal/metalloid stress tolerance [21]. AMF colonize the roots of most terrestrial plants [22], including ferns; once the symbiosis is established, they improve plant nutrition [23], modify root architecture [24], and promote plant tolerance or resistance to pathogens [25,26,27], drought [28], and salinity [29]. During phytoremediation, the use of AMF could positively affect the plant growth, mitigating the adverse effects of heavy metals and As or influencing their absorption, accumulation, and translocation [21,30].

It has been shown that plants growing on As-polluted soils are usually colonized by mycorrhizal fungi [31,32] and that the AMF hosted by the plant enhance As tolerance, mostly by restricting As uptake: this has been observed in many spermatophyta species, including *Holcus lanatus* L., *Medicago truncatula* Gaertn., *Helianthus annuus* L., *Lens culinaris* Medik., and *Plantago lancelolata* L. [33,34,35,36,37]. By contrast, some ferns, and especially *P. vittata* can form a symbiotic relationship with AMF that can increase the As accumulation in its fronds [4,14,15,18]. The knowledge about the relationship between *P. vittata* and AMF is limited to results obtained growing in controlled conditions and using single contaminants [18,38,39]. Therefore, considering that the phytoremediantion results are often related to lab scale experiments [40], with this work we tried to contribute to more extensive use of this technique in the field. For this purpose, we studied the As remediation efficiency of *P. vittata*, inoculated with a pool of AMF, firstly in pots containing soil coming from the site object of study, under controlled conditions for two years. Then, we successfully performed a three year field experiment in an industrial site (northwestern Italy) contaminated with As and several heavy metals.

## 2. Results

### 2.1. P. vittata Growth in Greenhouse: Experiment in Controlled Conditions

Table 1 shows the mean value of As and other metal concentrations in the batch of industrial soil used for the pot trial, compared to the limits established by the Italian law (Legislative Decree 152/06) [41]. As, Sb and Se levels were found to be higher than the permissible limits.

**Figure 1:**
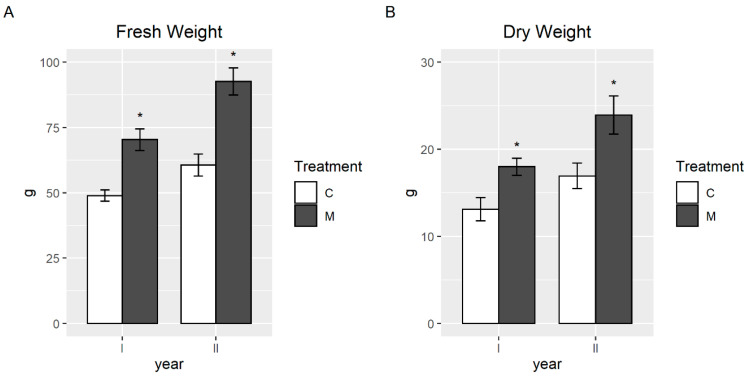
Average fresh (**A**) and dry weight (**B**) of aerial parts of *P. vittata* plants, inoculated (M) or not (C) with a mixture of arbuscular mycorrhizal fungi (AMF), in the pot experiment. The Asterisk (‘*’) indicates that the differences among the treatments in the same year were significant (*p*-value < 0.05). Error bars indicate standard errors.

Inoculation with AMF improved plant growth during the two years of the experiment performed in the greenhouse. In detail, in the first year, the fresh and dry weight of the aerial parts increased by 44% and 37%, respectively, in plants inoculated with selected AMF, compared to the uninoculated controls. In the second year, the fresh biomass of the AMF fronds increased by 52% and the dry one by 41% (Figure 1) compared to controls. The fresh biomass of uninoculated and AMF ferns increased by 24% and 32% in the first and in the second year, respectively (Figure 1A), while the dry biomass increased by 29% and 33% (Figure 1B).

#### 2.1.1. Arsenic Bioaccumulation in *P. vittata*

**Figure 2:**
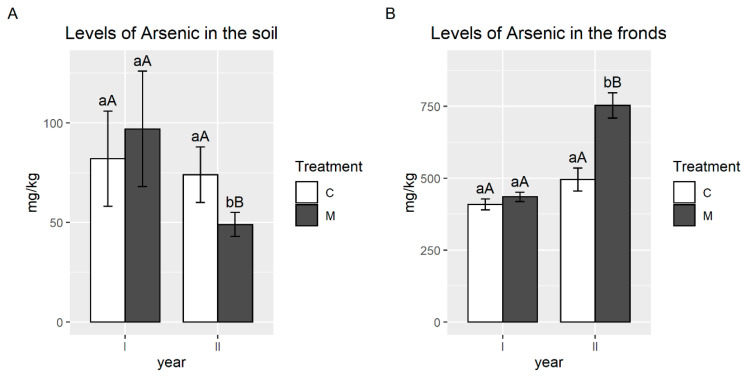
Arsenic average concentration (mg/kg) in soil (**A**) and aerial parts (**B**) of *P. vittata* plants, inoculated (M) or not (C) with a mixture of AMF, in the pot experiment. Different lower-case letters indicate a significant difference in the same year and different capital letters indicate a significant difference between the years (*p*-value < 0.05). Error bars indicate standard errors.

Before planting *P. vittata*, the level of As in the soil, was 170 mg/kg. During the first year of the trial, the concentration of As in soil was reduced by 51% (uninoculated controls) and 49% (AMF inoculated plants). Analyses performed after two years of plant growth showed that in the pots where AMF inoculated ferns were planted, the As levels slowed down the limit established by Italian Decree 152/06 (50 mg/kg) [41]. On the contrary, in the pots planted with uninoculated ferns, the As level was higher than 50 mg/kg (Figure 2A). In the first year, As accumulation in the fronds did not differ between AMF inoculated and uninoculated plants. The As concentrations were in the range 277–564 mg/kg d.w. (average value 409) in the uninoculated ferns and in the range 256–607 mg/kg d.w. (average value 435) in the AMF inoculated ferns. In the second year, the absorption of As was higher than in the first one: As frond content ranged from 137 and 855 mg/kg d.w. (average value 496) in uninoculated ferns and from 231 and 1235 mg/kg d.w. (average value 753) in AMF inoculated ones (Figure 2B). In the first year, the bioaccumulation factor (BF), defined as the ratio between As concentration in the plant tissue and As concentration in soil, was 2.4 and 2.5 in uninoculated controls and AMF inoculated plants, respectively. In the second year, BF increased to 6 in uninoculated controls, and 7.8 in AMF inoculated plants.

#### 2.1.2. Mycorrhizal Colonization

At the end of the two years, the intensity of the mycorrhizal colonization in AMF inoculated plants (M%) was about 50.9%. The uninoculated ferns were also colonized by AMF fungi but a lower level than AMF inoculated plants (M% about 10.1%).

### 2.2. In Field Experiment

Table 2 shows the mean value of As and other heavy metal concentrations in 18 soil samples harvested in the study area at two different depth compared to the limits established by the Italian law (Legislative Decree 152/06) [41]. As, Sb and Se levels were found to be higher than the permissible limits.

**Figure 3:**
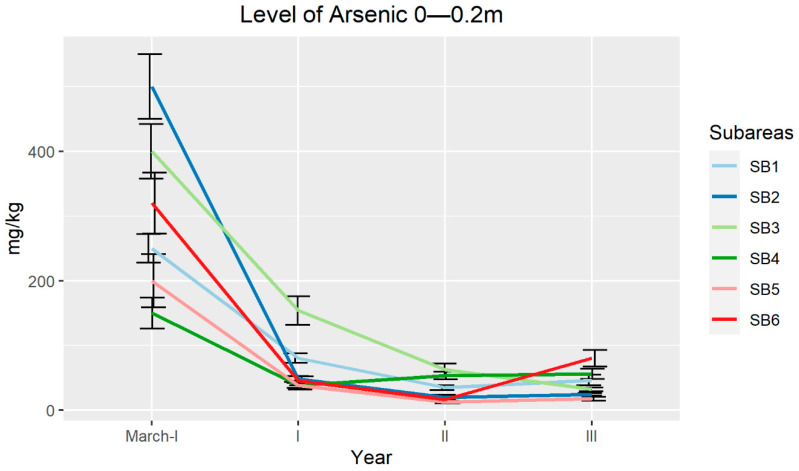
Arsenic average concentration (mg/kg) determined in soils during the three years of field trial at a depth of 0–0.2 m. Error bars indicate standard errors.

**Figure 4:**
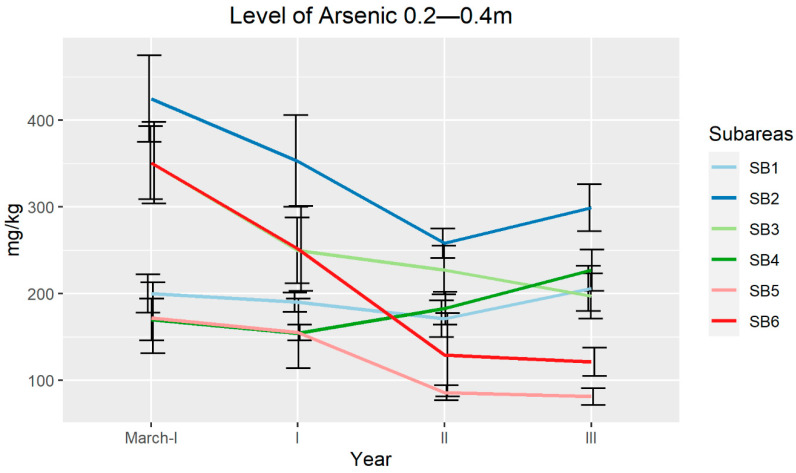
Arsenic average concentration (mg/kg) determined in soils during the three years of field trial at a depth of 0.2–0.4 m. Error bars indicate standard errors.

The chemical analyses on different subareas revealed a reduction in the As level in the soil at a depth of 0–0.2 m. In fact, at the end of the experiment, only subarea 4 (SB4) and SB6 showed values that were still above the limits set by the Italian law (Figure 3). As in the soil at a depth of 0.2–0.4 m showed a reduction not so marked as in the surface layer (Figure 4). While in the superficial layer the reduction was constant, in the underlying layer, the values were variable in the different years, with no SBs below the Italian law limits. Only SB5 reached an average value of 81 mg/kg after 3 years of experimentation.

**Figure 5:**
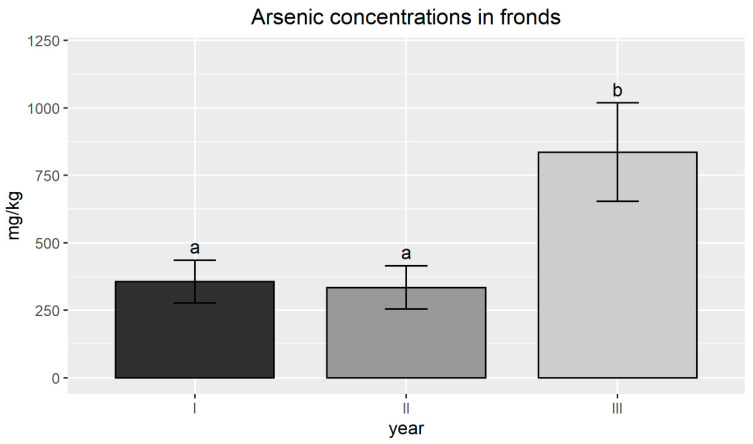
Arsenic average concentration (mg/kg) measured in *P. vittata* frond samples collected from the six subareas (SBs). Error bars indicate standard error and different letters indicates that the differences among the years were significant (*p*-value < 0.05).

The As uptake by *P. vittata* was highest in the third year of the field experiment: in the first and second year, the As uptake was similar, with concentrations ranging from 96–582 mg/kg d.w. (average value 355) and 177–711 mg/kg d.w. (average value 334). In the third year, the bioaccumulation was significantly higher than in the previous years, with concentrations in the range 397–1661 mg/kg d.w. (average value 835) (Figure 5).

**Figure 6:**
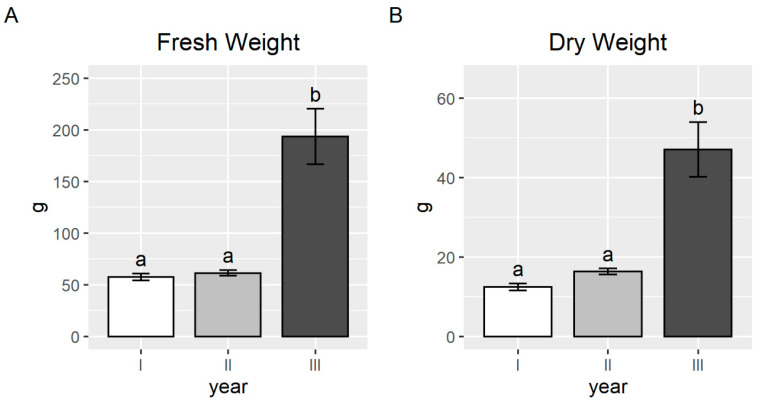
Average fresh weight (**A**) and dry weight (**B**) of *P. vittata* aerial parts in the pot experiment. Error bars indicate standard error and different letters indicates that the differences among the years were significant (*p*-value < 0.05).

The ferns’ growth in the field conditions was similar during the first two years of the experiment. However, in the third year, the plant biomass significantly increased. The fresh weight increased by 50 g to 200 g and the dry weight increased by 15 g to 48 g (+225% for both weights), with a high number of fronds and an average height of 86 cm (range values 78–97, data not shown) in the third year (Figure 6).

In the first year, the BF calculated for the depth 0–0.2, ranged between 1.2 and 11.9 (average value 6.5). In the second year increased to 6.9–14.2 (average value 10.9) and in the third year reached 12.1–29.7 (average value 21.2).

## 3. Discussion

The As concentration in the soil of the considered industrial site exceeded the limit imposed by the Italian D.lgs 152/06, indicating a potential human health risk [41]. Therefore, bringing the concentration values of As into the soil within the limits established by law was mandatory. Several physical and chemical methods can be applied in order to reduce the As level in soil, but the high cost of these procedures have often limited their application to small-scale operations [42]. Phytoremediation in the last two decades has emerged as a potential in situ technique at low-cost [11] but, despite the excellent results obtained in the lab by numerous scientists, the scientific literature documenting its application in the field is still rather scanty [40]. Anyway, some of these report the effect of *P. vittata*, used in the field [4,43,44,45], or as an intercropping plant [46,47]. However, none of these papers focused on the phytoremediation activity of AMF inoculated fern. To our knowledge, only Chen [48], studied the effects of AMF on *P. vittata* grown in a uranium mining-impacted soil, profoundly different from the site object of our investigation.

In this work, we studied the As remediation efficiency of *P. vittata* inoculated with AMF in a highly heavy metal polluted site.

The study was performed in two steps. In a first 2 year experiment performed under controlled conditions, plants were inoculated or not with AMF and cultivated in a greenhouse on a multi contaminated soil coming from the industrial site used for the in situ remediation. In the second experiment we evaluated the capability of AMF-inoculated *P. vittata* ferns to accumulate arsenic in situ for three years. The results obtained during the pot trial showed that initially the positive effects of the selected AMF pool were only on the plant growth and health. In the second year, the AMF induced positive effects also in the bioaccumulation factor: AMF inoculated ferns showed the highest accumulation of As, with a reduction in the metalloid in the soil from 170 to 49 mg/kg. These results are in agreement with other works [15,48,49,50] demonstrating that phytoremediation can be increased by inoculating *P. vittata* with selected AMF. In fact, AMF play a key role in the mobilization/immobilization of metal ions and could alter their availability to plants [21]. AMF may lead to protective effects through changes in metals solubility mediated by changes in the soil solution pH, or by immobilizing heavy metals in and on fungal biomass [51] Moreover, ferns inoculated with the AMF *Funneliformis mosseae* exhibited higher activities of antioxidant enzymes [49] and up-regulation of multiple forms of glyceraldehyde-3-36 phosphate dehydrogenase, and other enzymes playing a central role of glycolytic enzymes in arsenic metabolism [18]. Low levels of mycorrhizal colonization occurred in the control ferns grown in polluted soils for two years. The pot experiment confirmed the possible occurrence of indigenous AMF adapted to the polluted environment. This is consistent with the literature reporting the presence of AMF able to grow in heavy metal polluted soils. Turnau and co-workers [52] showed that AMF isolates in an old zinc wastes decreased heavy metal uptake by plants growing on metal rich substrata. In another study, Leung and co-workers highlighted the occurrence of indigenous AM fungi inside the roots of *P. vittata* growing in mining sites; in this case, the concentrations of As in fronds were 24-fold higher than in roots [53]. Finally, Baghaie and co-workers [54] reported that indigenous AMF reduced Cd uptake. Anyway, despite the colonization of indigenous AMF, the best effects on As accumulation and on plant growth were obtained from the ferns inoculated with our pool, that induced a significantly higher colonization degree.

The chemical characterization of the soil of the industrial site showed that As is associated to oxides/hydroxides of Fe and Mn (data not shown); Bettiol and coworkers [4] reported that in a similar soil, reducing or acidic conditions could make As bioavailable. The production of root exudates, containing various compounds including phytic acid and oxalic acid, can also mobilize As [55].

The results obtained during the in situ trial were similar to those of the pot experiment. In the field, both plant biomass and As accumulation were highest in the third year, probably due to the reduction in As content in the surface layer. The trend of the decrease in the metalloid in soil was similar to that observed in the pot experiment. After three years of growth, the As amount in almost all SBs was below the limits prescribed by the Italian Decree 152/06, except for SB6 (average 81 mg/kg) at the depth 0–0.2 m. Concerning the depth of 0.2–0.4 m, As levels declined slowly, but still remained high compared to the limits prescribed the Italian Decree 152/06. Our results disagree with [43]; they showed that the greatest As decrease in the soil was found in the 0.15–0.30 m depth, under the root zone. The reduction in the absorption of As in-depth 0.2–0.4 m could be related to the level of Cd: as reported by Balestri and co-authors [56], the highest concentration of Cd that *P. vittata* can tolerate is 6.7 mg/kg. Chemical analysis on the industrial site’s soil showed an average Cd concentration of around 7 mg/kg. Cd level could, therefore, had led to changes at the root level that inevitably limited and altered the root’s functions, with a possible reduction in the absorption of As.

## 4. Materials and Methods

### 4.1. Fern Propagation and Growth

Plants of *P. vittata* were propagated from spores collected in Genova (Italy) [15]. After 1 month, gametophytes grown on Petri dishes by germinating spores (see Supplementary Figure S1) were transferred into sterile polyethylene boxes (Duchefa Biochemie B.V., Haarlem, The Netherlands), on vermiculite moistened with Murashige-Skoog mineral solution (1/10 strength macronutrients + micronutrients) for sporophyte production.

Three months after spore germination, the sporophytes were transferred in 0.5 L black polyethylene pots filled with quartz sand (0.2–0.7 mm coarse grade) (see Supplementary Figure S2). Plants were fed three times for week with 100 mL of Long Ashton nutrient solution [57] containing 32 µM phosphate (added as NaH_2_PO_4_·2H_2_O) and watered with deionized water when needed.

### 4.2. Experiment in Controlled Condition

One-year-old sporophytes were divided into two batches: 10 sporophytes (M) were inoculated with sporocarps, spores, hyphae of AMF belonging to the genera *Rhizophagus and Funelliformis*, and root fragments (collected from agricultural soil, produced and provided by Mybasol S.r.l., Alessandria, Italy), while 10 were uninoculated and used as control (C). After two months, the plants were transferred in 25 L polyethylene pots, filled with soil collected in the industrial site (from a batch of 500 L). For two years, the plants grew in a greenhouse with 30% shading, and each year harvested in November. Aerial parts were weighed and processed for Inductively coupled plasma-optical emission spectrometry analyses (ICP-OES).

#### Mycorrhizal Colonization

Root portions were fixed in 70% ethanol, stored at 4 °C and cut in forty 1 cm long pieces. After clearing with 10% KOH for 45 min at 60 °C, root pieces were stained with 1% methyl blue in lactic acid, and mounted on a slide. Mycorrhizal colonization was estimated according to Trouvelot and co-authors [58].

### 4.3. Field Experiment: Study Site and Phytoremediation Design

**Figure 7:**
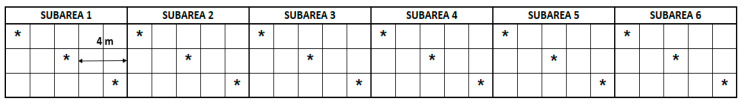
Field area divided into six subareas (SBs). The Asterix (‘*’) indicates the sampling point for soils collected at the end of each year.

The study area was located in northwestern Italy and was contaminated with heavy metals due to the metallurgic planting facility’s activities, dismantled in the early 1980s. In March 2014, an area 60 × 1.5 m was set up and divided into six subareas (SB) (Figure 7). Mycorrhized sporophytes were planted: their density was five sporophytes per sq m; among the sporophytes (see Supplementary Figure S3), the soil was covered with a black plastic sheet to reduce the autochthonous vegetation development. Thirty days before planting, the experimental site was treated with Glyphosate (25 mL/L) to remove the weeds and tilled. A sheet of 30% shading covered the ferns during the summer. In November, we collected each soil (at two different depths, 0–0.2 m and 0.2–0.4) and aerial parts of *P. vittata*; the concentrations of As both in soil and fronds were measured. During the winter, some small tunnel greenhouse protected the ferns (see Supplementary Figure S4). The experiment was carried out for three years.

### 4.4. Soil Sampling and Determination of Arsenic and Heavy Metals in Soil

The soil tested for phytoremediation consisted of filling materials (0–0.7 m below ground level). Core samples were collected for both experiments: for the pot experiment, one soil core (2 cm in diameter) was taken over the entire depth for each pot, for the field experiment three soils cores (5 cm in diameter) were taken from each SBs at two depths (0–0.2 m and 0.2–0.4 m below ground level). Core samples were collected at planting time and at harvesting time (November), freeze-dried, homogenized and analyzed for As and metals (Cd, Cr, Cu, Fe, Mn, Ni, Sb, Se, Sn, Zn). For total concentration analysis, 0.1 g-aliquots of soil samples were microwave digested with sample preparation system MDS 2000 (CEM, Indian Trail, NC, USA), with 8 mL of aqua regia/HF mixture for 60 min at T = 170 °C, in according to Bettiol and co-workers [4]. Analytical determination of As and metals in the solutions obtained after each extraction and after total digestion was carried out by ICP-OES, using a JY24 (Jobin-Yvon, France) spectrometer equipped with AT5000 + ultrasonic nebulizer (Cetac Technologies, Omaha, NE, USA.

### 4.5. Arsenic Determination in P. vittata

Samples of *P. vittata* leaves were collected at the harvesting time (November), according to our previous results [14] to avoid As leaching. The leaves were carefully washed with deionized water, freeze-dried, and homogenized. High purity reagents were used for sample digestion. Aliquots of about 0.1 g of plant tissue samples were treated with 65% nitric acid (5 mL) in 25 mL glass flasks fitted with air-cooled condensers. Solutions were heated at 140 °C for 3 h. As concentration was measured by ICP-OES. For all determinations, the wavelength of 193.759 nm was used. Aqueous standard solutions with As concentrations up to 10 µg/mL were used for calibration, and samples exceeding this limit were diluted.

### 4.6. Statistical Analysis

R (version 4.0.0, CRAN, package: userfriendlyscience) was used for statistical analysis. The normality of the samples was evaluated with Shapiro–Wilk’s method (package: rstatix), followed by Bartlett’s test for homogeneity of variances (package: stats). One-way or two-way analysis of variance (ANOVA) was followed by a Tukey–Kramer multiple comparisons test. The significance level was set at *p*  <  0.05.

## 5. Conclusions

In summary, our results suggest that the phytoremediation of the metallurgic site would require at least four years to reduce the concentration of As to 50 mg/kg in agreement with the Italian Law (for industrial areas). Besides, *P. vittata* plants’ inoculation with selected AMF is necessary to allow their growth in such polluted soil. In follow-up investigations, we will screen other plants’ use to reduce the level of heavy metals. The research on the soil ecology of the industrial site could expand our knowledge of phytoremediation.

## Figures and Tables

**Table 1:** Average concentration (mg/kg) of metals in the industrial soil. Number of replicas 3.

	Mean ± SE	Limits by Italian Law for Industrial Areas
As	170 ± 31	50
Cd	2 ± 0.7	15
Cr	401 ± 37	800
Cu	221 ± 14	600
Fe	82,726 ± 11,258	-
Mn	301 ± 71	-
Ni	82 ± 6	500
Sb	33 ± 5.2	30
Se	29 ± 7.2	15
Sn	15 ± 3	300
Zn	428 ± 69	1500

**Table 2:** Average concentration (mg/kg) of heavy metals in the soil at two depth sampling points in the study area. Number of replicas 18. [a] mean ± SE.

	Soil Depth 0–0.2 m [a]	Soil Depth 0.2–0.4 m [a]	Limits by Italian Law for Commercial Area
As	230 ± 80	270 ± 72	50
Cd	5 ± 1	7 ± 0,1	15
Cr	492 ± 30	553 ± 70	800
Cu	248 ± 34	167 ± 39	600
Fe	84,667 ± 12,831	56,000 ± 6398	-
Mn	389 ± 11	743 ± 32	-
Ni	104 ± 6	105 ± 3	500
Sb	37 ± 8	15 ± 3	30
Se	21 ± 7	7 ± 2	15
Sn	15 ± 3	10 ± 2	300
Zn	572 ± 95	303 ± 49	1500

## Supplementary Materials

The following are available online at https://www.mdpi.com/2223-7747/9/9/1211/s1, Figure S1: gametophytes grown on Petri dishes, Figure S2: sporophytes on pots filled with quartz sand. Figure S3: sporophytes planted on field. Figure S4: tunnels covering the ferns during winter.
