# Supplementary figures and images for: Phytoremediation of a Highly Arsenic Polluted Site, Using Pteris vittata L. and Arbuscular Mycorrhizal Fungi
Source: Plants (Basel). 2020 Sep 16;9(9):1211. doi: 10.3390/plants9091211 (PMC7570287; doi:10.3390/plants9091211)

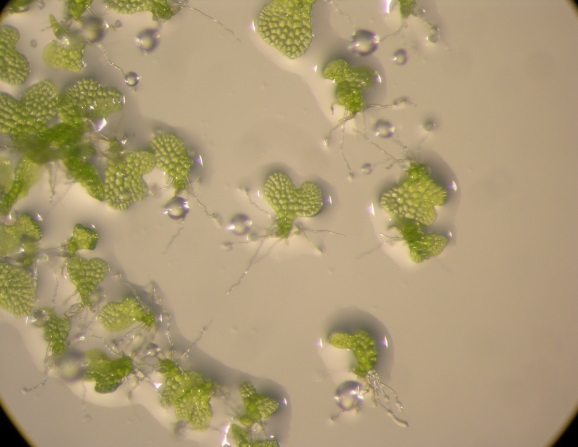


**Figure S1.** Gametophytes grown on Petri dishes 30 days after spore germination

Supplement: Supplementary file 1 [file plants-09-01211-s001.zip › Figure S1.docx]

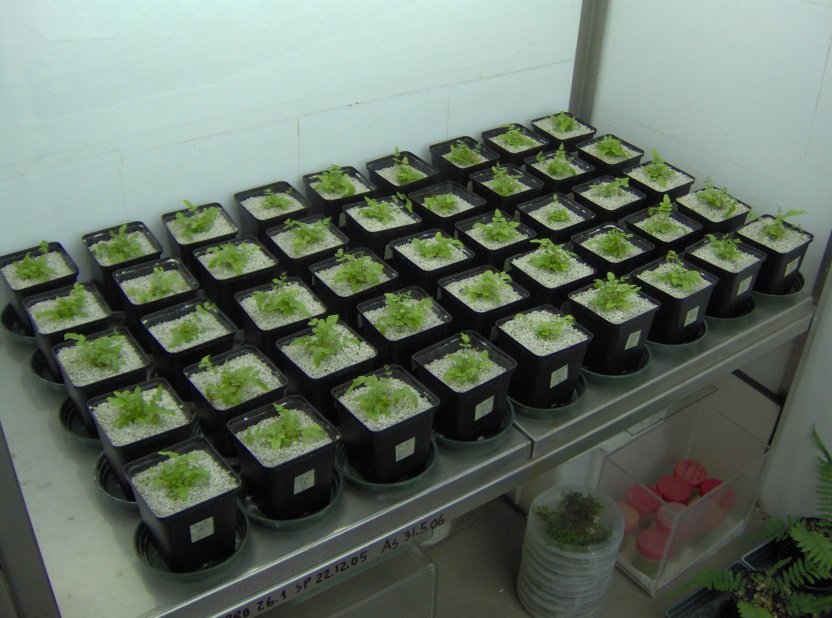


**Figure S2.** Sporophytes on pots filled with quartz sand.

Supplement: Supplementary file 1 [file plants-09-01211-s001.zip › Figure S2.docx]

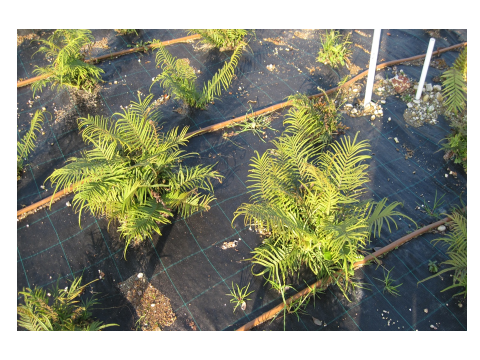


**Figure S3.** Sporophytes planted on field.

Supplement: Supplementary file 1 [file plants-09-01211-s001.zip › Figure S3.docx]

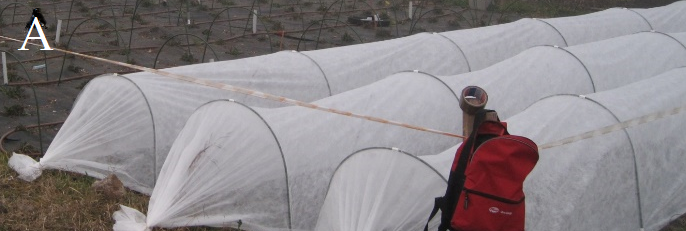


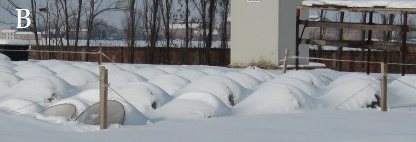


**Figure S4A-B.** The tunnels during winter, covered or not with snow

Supplement: Supplementary file 1 [file plants-09-01211-s001.zip › Figure S4.docx]
